# April Fooled

**Published:** 1856-04

**Authors:** 


A story is told in Nelson's Lancet of a brother of the profession, who was most laughably *sold* in Buffalo, by a boy early in the morning of April last. The Doctor was aroused about daylight, by strong pulls at the door bell, and after poking his head out of the window, which he had raised to see what was wanted, he enquired whether any body had the *cholera*, *small-pox*, or something else, but when the little fellow said that his *father said*, "there was to be a partition in the family," he understood at once the whole matter, and reluctantly prepared to go, for the morning was cold, and the snow was on the ground. Streets, alleys, lanes, by-ways, snow-drifts, and mud holes were passed, until the Doctor begged leave to rest awhile, and sent the boy to tell his mother he was coming. There he sat awaiting the return of the boy, who took good care not to do so, when cold and shivering, our brother turned for home. Whilst at breakfast, and running over the events of the morning in his mind, some one discovered *chalked* in legible letters on the back of his coat, "April Fooled." Our brother will require a *bond*, signed, sealed, and delivered, before he goes abroad another *April* morning.

Now this was fun for the boy and taught the Doctor a lesson of caution, which each return of April may call to his memory. But unfortunately, doctors are made fools of every day in the year, by persons much less innocent in their designs, than the youthful wag alluded to. Through every vicissitude of weather, they are run off of their legs to wait upon the sick, who always regard a demand for a bill as an attempt at a hoax, or direct attack upon their intellect, for they think none but fools pay Doctors.

